# The positive impact of daily well-being practices on individual veterinary professionals’ professional quality of life self-assessment scores within an emergency and specialty hospital

**DOI:** 10.3389/fvets.2024.1381090

**Published:** 2024-10-08

**Authors:** Ames J. Alwood, DIana A. Ferrentino, Sonja A. Olson, Veronyca I. Rodriguez

**Affiliations:** ^1^Authentic DVM, Longmeadow, MA, United States; ^2^Health & Well-being Team, BluePearl Specialty + Emergency Pet Hospital, Tampa, FL, United States; ^3^Heartstorming Wellness LLC, Vancouver, WA, United States; ^4^Consultant, Philadelphia, PA, United States

**Keywords:** well-being, ProQOL, burnout, compassion fatigue, compassion satisfaction, veterinary professionals, veterinarians, occupational stress

## Abstract

**Introduction:**

Our study set out to identify the benefits for veterinary workers of structured daily well-being practices on compassion satisfaction, burnout, and secondary traumatic stress. Suggested origins of high rates of psychological stress and burnout are significant occupational concerns for veterinary workers. Many professional helpers experience an extreme state of tension and preoccupation from exposure to the suffering of those being helped. Veterinary workers are further impacted by negative associations and moral distress experienced due to limitations in the provision of quality medical care for veterinary patients. These negative experiences exist despite veterinary professionals’ work being worthwhile and highly valued.

**Methods:**

A randomized controlled study was performed over 6 months. Volunteer participants were members of a team of veterinary workers at a 24-h emergency and specialty hospital. Study participants were asked to incorporate daily well-being practice(s) into existing routines whereas control participants were not. Measures of well-being and the negative impacts of serving as veterinary helpers were assessed by having participants complete the Professional Quality of Life (ProQOL) self-assessment at baseline and at 1, 3 and 6 months. Composite scores for compassion satisfaction, burnout and secondary traumatic stress were calculated.

**Results:**

Baseline ProQOL scores were similar between study participants and controls. Baseline compassion satisfaction (CS), burnout scores (BS), and secondary traumatic stress (STS) scores for those instituting well-being practices were 37.6 (+/− 3.6), 26 (+/− 5.3), and 26.6 (+/− 5.2). Study participants had higher compassion satisfaction scores at 6 months with mean CS scores (*n* = 15) of 40.1 (+/− 6.8) and adjusted scores that were on average 3.0 (95% CI 0–6.1) higher than the control group (*p* = 0.048). Significant differences in BS or STS scores were not seen.

**Discussion:**

Improvements were seen in CS but not BO/STS for those caregivers who implemented well-being practices into their daily routines. Factors that likely contributed to successful implementation of well-being practices include educational resources, supportive leadership, accessibility, and consistent acknowledgement and positive rewards. Proposed supportive elements and resources for maintenance of well-being practices within a team of veterinary workers include provision of a psychologically safe community and team support (including formal or informal “buddy systems”).

## Introduction

Veterinary medicine professionals’ (Appendix 1) well-being has become an area of growing focus in recent years. The impact of “practice-related stressors” on veterinarians has been recognized to contribute to psychological distress, risk of suicidal ideation, and related challenges to mental health and well-being (1–5). Similar impact is noted to result in increased burnout, emotional exhaustion, and decreased professional efficacy among veterinary technicians (6, 7). New stressors: including a lack of resources, increases in economic hardship, vicarious trauma, psychological distress, and loneliness impacted individuals, communities, and the veterinary profession over the last few years. This was magnified by the COVID-19 Pandemic which brought new challenges such as curbside service which separated families from pets and care teams from families, and new concerns for team safety (e.g., risk of COVID related illnesses). As the pandemic progressed, there was also a recognized need for each hospital to intermittently pause service provision due a heightened imbalance of caseload and caregiving abilities (e.g., helper fatigue and absolute limitations in the number of staff available to provide care). A significant increase in compassion fatigue and burnout in the veterinary industry worldwide is suspected to be a result of these stressors (8) and recently has been proposed by the research of Ouedraogo and Lefebvre (9).

The Professional Quality of Life (ProQOL) self-report assessment tool has been used over the last 20 years in many behavioral science studies in both human and veterinary environments. The ProQOL was developed originally by Beth Hudnall Stamm, PhD, to be used for human health care providers. The tool is utilized to gage helper well-being by assessing compassion fatigue, compassion satisfaction, and level of burnout at a point in time. The AVMA lists it as a resource for veterinarians to assess their quality of life, specifically how one feels in relation to their work as a helper (10). Veterinary care workers function at the intersection of animal patients and their guardians. Both positive and negative aspects of caregiving can influence an individual’s professional quality of life, therefore this assessment measures compassion satisfaction (the “positive”) as well as compassion fatigue (the “negative”) to provide a starting point for self-reflection about both the individual and their environment. By extension, ProQOL can help identify areas of focus for self-care and support the development of individualized mindful interventions.

It is well known that there is not enough published research to support the impact of well-being initiatives on veterinary care teams. The impact of mindfulness practices, gratitude, reframing, and overall stress reduction techniques on the health and well-being of veterinary caregiving professionals is an area of growing interest. While such well-being practices are evidence-based in human medicine and related caregiving environments (11), research in veterinary medicine is not yet broadly accepted as important or valuable. Additionally, practices of well-being, and the prioritization of associate health and well-being, requires a “person-first” mentality that may at times feel in conflict with common clinical and business priorities of patient and client care. Increasing the prevalence and diversity of veterinary well-being initiatives, while critically assessing their impact with outcome studies such as the one we conducted, will provide valuable contributions to the growing body of knowledge and research on what is beneficial for our veterinary care teams.

In 2022, we conducted a study to investigate the impact of daily well-being practices on a team of veterinary care workers. We hypothesized that an associate-driven and management-endorsed well-being initiative that actively involves veterinary helpers will result in improved psychological well-being and workplace satisfaction as measured by the ProQOL self-report tool.

## Materials and methods

### Study design

A prospective longitudinal study was performed from February 2022 through August 2022. Recruitment of volunteer study participants was initiated in the Fall of 2021 and concluded in January 2022. Participants were members of a team of veterinary helpers at a 24-h emergency and specialty hospital that serves a diverse population of pets and families in a suburb of the metropolitan areas of Wilmington, Delaware and Philadelphia, Pennsylvania. The veterinary team included a total of 21 veterinarians, 46 veterinary technicians and 18 assistants and an available ECC staff sample size of 12 veterinarians and 30 veterinary technicians and assistants.

In September 2021, one or more ECC veterinarian(s), (including the study investigator Alwood) representatives of the Health + Well-being team, and the management team facilitated an introductory meeting and discussion group of all interested employees and volunteer study participants. Study participants were enrolled on a volunteer basis from the hospital team. Recruitment and enrollment of volunteers was enhanced by information shared at team meetings, the creation of virtual and traditional flyers highlighting the opportunity for the local hospital team, and direct in-person communication with employees from the study investigator, social workers and management team. The study was funded by a nationally awarded grant which allowed for participants to be individually rewarded by periodic delivery of “DoorDash” gift cards upon completion of their confidential ProQOL surveys. Participants included veterinarians, veterinary technicians, support staff and on-site administrators.

The Professional Quality of Life assessment tool (ProQOL) (see Appendix 2) utilizes 30 standard questions which provide composite scores in compassion satisfaction (CS), burnout (BS), and secondary traumatic stress (STS).^1^ Each score has a potential maximum score (raw score) of 50. Compassion satisfaction (CS) relates to the positive aspects of helper work, where a higher CS score indicates greater job fulfillment. BS and STS each represent manifestations of the negative consequences of helping with scores above 41 indicating a need to evaluate causes of negative feelings or experiences related to work environments or exposures.

Each study participant was asked to complete a baseline ProQOL prior to adding daily well-being practice(s; WB) to their personal activities. The practices could be either (1) Utilizing Lyra (free mental health online support platform for BluePearl associates), (2) a daily practice of gratitude, (3) a breathing exercise (examples provided), or (4) a combination of these practices (see Appendix 3). Study participants were asked to continue such practices throughout the 6-month study period and were asked to complete ProQOLs at one, three and 6 months. A control group consisted of a cohort of volunteer hospital associates who completed ProQOLs at identical timepoints but were not asked to add WB to their daily activities. The control cohort, while not asked to add any new WB, was not prohibited from participation in any well-being activities.

### Study methods

Study participants were asked to complete the ProQOL questionnaire and share the survey (via email) with a co-investigator Social Worker without direct interactions with participants (Ferrentino). Each survey was blinded by this Social Worker using a randomly assigned participant identification number. The blinded surveys for both study participants and control cohort members were then forwarded to a co-investigator who entered results into an Excel spreadsheet. Survey results were entered into the data analysis spreadsheet and composite scores for compassion satisfaction (CS), burnout (BS), and secondary traumatic stress (STS) were calculated.

Following completion of the baseline ProQOL, study participants were then asked to choose one, or a combination of, WB to add to their daily work-life. Study participants were asked to continue daily WB for the six-month duration of the study. They were asked to complete new ProQOL surveys at one, three and 6 months. Each survey was processed as the baseline survey(s) had been and only completed surveys were included in data analysis. DoorDash gift cards were utilized as an incentive for participants (study and control group members) to complete surveys at baseline, one and 6 months.

Throughout the duration of the study, participants were encouraged to continue the daily WB by various mechanisms. Such mechanisms included the efforts of established Health and Well-being Ambassadors (independent of the study investigators), a semi-weekly on-site Social Worker presence (co-investigator) to offer virtual support and conduct needs assessments related to wellness. Social Worker along with the Health and Well Being team helped to identify, designate, and supply a safe and private space to process and access resources. Other mechanisms included monthly email reminders and support offering from the co-investigator specifically tasked with this role, and email communications referenced as “Wellness Wednesdays.” “Wellness Wednesdays” consisted of reminders and requests for each study participant to share activities chosen in the prior week as well as participant sharing of tools for success and/or perceived barriers for the completion of daily well-being practices. Additional support and facilitation were provided by monthly email check-ins from an additional co-investigator and Health and Well Being educator. Reminders were provided via email and via posted flyers prior to the next ProQOL survey date. Direct requests were made to individuals as needed.

### Statistical analysis

All analyses were performed using SAS 9.4 (SAS Institute Inc.; Cary, NC). A significance threshold of 0.05 was used. Data was analyzed to evaluate differences in scores over time and for comparison of study and control groups.

Linear mixed models were used to test for effects of group on ProQOL scores. The models had fixed factors of group, time, and a group by time interaction effect. A baseline covariate and a random intercept were determined for each participant. Model assumptions (normality and homoscedasticity of model residuals) were confirmed via inspection of QQ-plots and histograms of conditional residuals, and conditional residual plots. Simple effects of group(s) at 1, 3, and 6 months were tested. Satterthwaite degrees of freedom method and REML estimation were used.

As a sensitivity analysis, an alternate analysis was run with the change from baseline scores as the dependent variable and no baseline covariate. All other model specifications and comparisons were the same.

## Results

### Study enrollment and ProQOL survey completion over time

The planned study design was to enroll 45 volunteers and randomly assign 30 participants to a study cohort and 15 participants to a control cohort. Forty-five volunteers were identified initially (thus achieving the desired 30 to assign to the study cohort). One study participant withdrew before study initiation (before receiving a baseline survey for completion). An additional four study participants did not submit baseline ProQOLs and did not proceed with any subsequent study requirements. One study participant’s baseline survey was incomplete and thus excluded from data collection. Thus, a total of 24 baseline ProQOL were available from the original 30 in the study group.

Two additional study participants did not submit a one-month ProQOL, thus 22 ProQOL surveys were available for review and comparison at 1 month. It was elected to only include the data from these remaining 22 participants when reporting baseline and 1 month study participant survey results.

Of the initial 15 participants assigned to the control cohort, one control participant was excluded due to potential conflict of interest. In addition to the control participant excluded prior to study initiation, two control participants did not submit baseline ProQOLs and did not proceed with any subsequent ProQOL survey completion(s). Thus, a total of 12 control ProQOLs were available for baseline and one-month review. Two additional individuals within the control cohort did not provide additional data after 1 month and one additional survey was not provided at 3 months. This resulted in a total of nine ProQOLs for the control cohort at 3 months but 10 surveys at 6 months.

Twenty-one participants provided ProQOL surveys for each study timepoint (12 study participants and nine control cohort members); an additional four participants completed the study but failed to submit a ProQOL survey at the three-month timepoint (one control cohort member and three study participants); two study participants (one due to a change in employment status) did not submit surveys after the three-month timepoint. Completion rates for surveys declined from baseline to 6 months. Only complete PROQOLs were analyzed.

**Figure 1:**
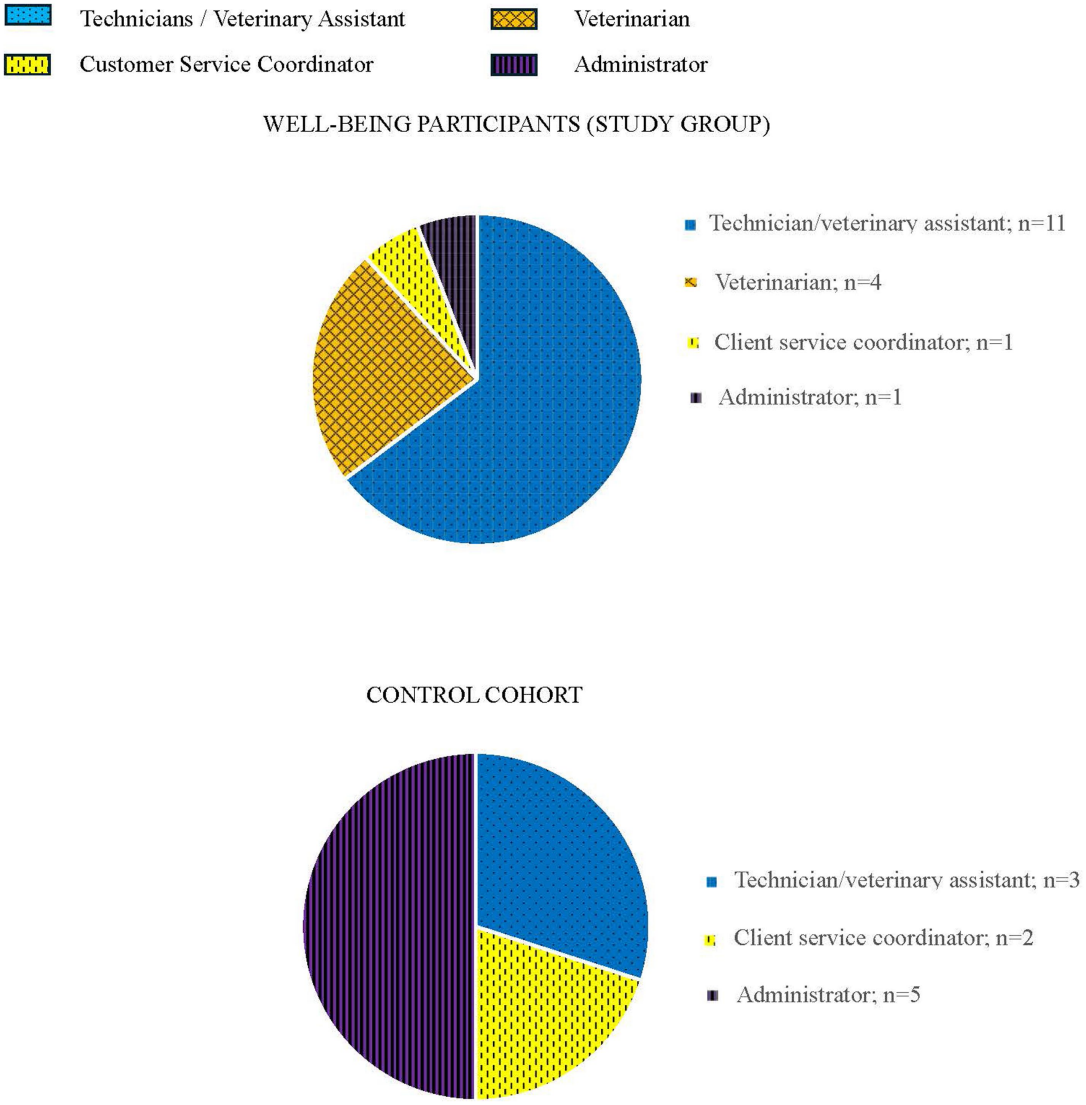
Composition of study and control groups by job category.

Figure 1 demonstrates the composition by job category of the 27 participants within study and control groups at study conclusion (*n* = 17 for remaining study participants and n = 10 for remaining control participants).

### Subjective findings

Email communication between study mental health associates (e.g., social worker and mental health educator) and study participants occurred on a weekly cadence (at minimum; e.g., “Wellness Wednesdays”). These communications provided additional information about participant experiences and the WB and their impact. Participants in the study commented that WB became a habit with practices performed without a need to intentionally think about it times of stress. Many participants cited improvements in reduction of stress and ease of work due to WB (e.g., “work has been easier”). Participants often established a routine for WB at set times of the day (e.g., beginning and end of day, or on regular break times) but also reported pausing to utilize WB during times of stress. Others shared that they adopted practices akin to a “Buddy system” to facilitate maintenance of WB or expanded their knowledge and awareness of WB to others (i.e., educating and advocating for the adoption of WB by those outside of the study).

Some participants reported utilization of technology to enhance WB, particularly there was common mention of utilizing breathing apps on personal smart watches. Another site-specific factor that likely enhanced performance of WB was the presence of a dedicated space for workers to practice mindfulness. The clinic team independently worked with management to create a space separate from the clinical areas (and hence the auditory and other sensory stimuli) which was dubbed the “cozy corner” or “Zen space.” This worker space provided soft seating, mats for stretching or yoga, dim lighting, meditation and relaxation tools and guides. More than one participant communicated an intentional division of their break times into part nutrition and part “cozy corner” time.

With respect to breathing exercises as a WB, more than one participant reported utilizing 4–7-8 breathing to enter sleep and thus the breathing exercise(s) likely enhanced sleep hygiene (a commonly reported health concern for veterinary workers). However, some participants also reported the adoption of new or alternative WB (especially breathing exercises) when either one became “less effective” (i.e., replacing 4–7-8 breathing with an alternative breathing exercise) over time or when an initial breathing exercise simply was not effective for the individual (e.g., some participants expressed increased feelings of anxiety with that breath technique).

Voluntary email communications and direct conversations with the co-investigator providing monthly “check-ins” with participants also identified challenges and/or barriers associated with completion of WB practices. Participant experiences included descriptions of actual and perceived time scarcity preventing or inhibiting success with WB. Some participants specifically reported feelings of fatigue and low energy could prevent them from completing WB. Additionally, helpers within the participant cohort described that the need to perform WB felt “like another burden.”

### Objective findings

Baseline ProQOL scores were similar between study participants and controls (Table 1). Baseline CS, BS, and STS scores [mean (+/− SD)] for those instituting WB were 37.6 (+/− 3.6), 26 (+/− 5.3), and 26.6 (+/− 5.2).

**Table 1:** Baseline PROQOL scores.

Analysis variable: score							
*VarName*	*TIME*	*GROUP*	*N*	*Mean*	*Std Dev*	*Minimum*	*Maximum*
Compassion Satisfaction	0	Control	12	37.3	3.8	31.0	43.0
		Intervention	22	37.6	3.6	31.0	43.0
Burnout	0	Control	12	22.9	3.6	18.0	29.0
		Intervention	22	26.0	5.3	17.0	36.0
Secondary Traumatic Stress	0	Control	12	21.5	5.6	15.0	32.0
		Intervention	22	26.6	5.2	19.0	40.0

The study group had higher compassion satisfaction scores than the control group (nearly significantly with *p*-value = 0.051) at 6 months. Mean CS scores (n = 15) of 40.1 (+/− 6.8) for the study group at 6 months. The study group had adjusted scores that were on average 3.0 (95% CI 0–6.1) higher than the control group. In a sensitivity analysis, analysis of the change scores did find this effect to be marginally significant (*p* = 0.048).

An increase in STS scores was seen at 1 month in the study group (*p* = 0.029) with adjusted scores that were on average 2.7 (95%CI 0.3–5.2) higher than the control group. In a sensitivity analysis, analysis of the change scores did not find this effect to be significant (*p* = 0.581) with the intervention scores only on average 0.8 higher (95%CI 2.0 lower −3.5) than the control group. This discrepancy makes the difference between these groups ambiguous or uncertain. The trend did not persist when analyzing differences at three and 6 months. No effect on BO scores was seen.

**Figure 2:**
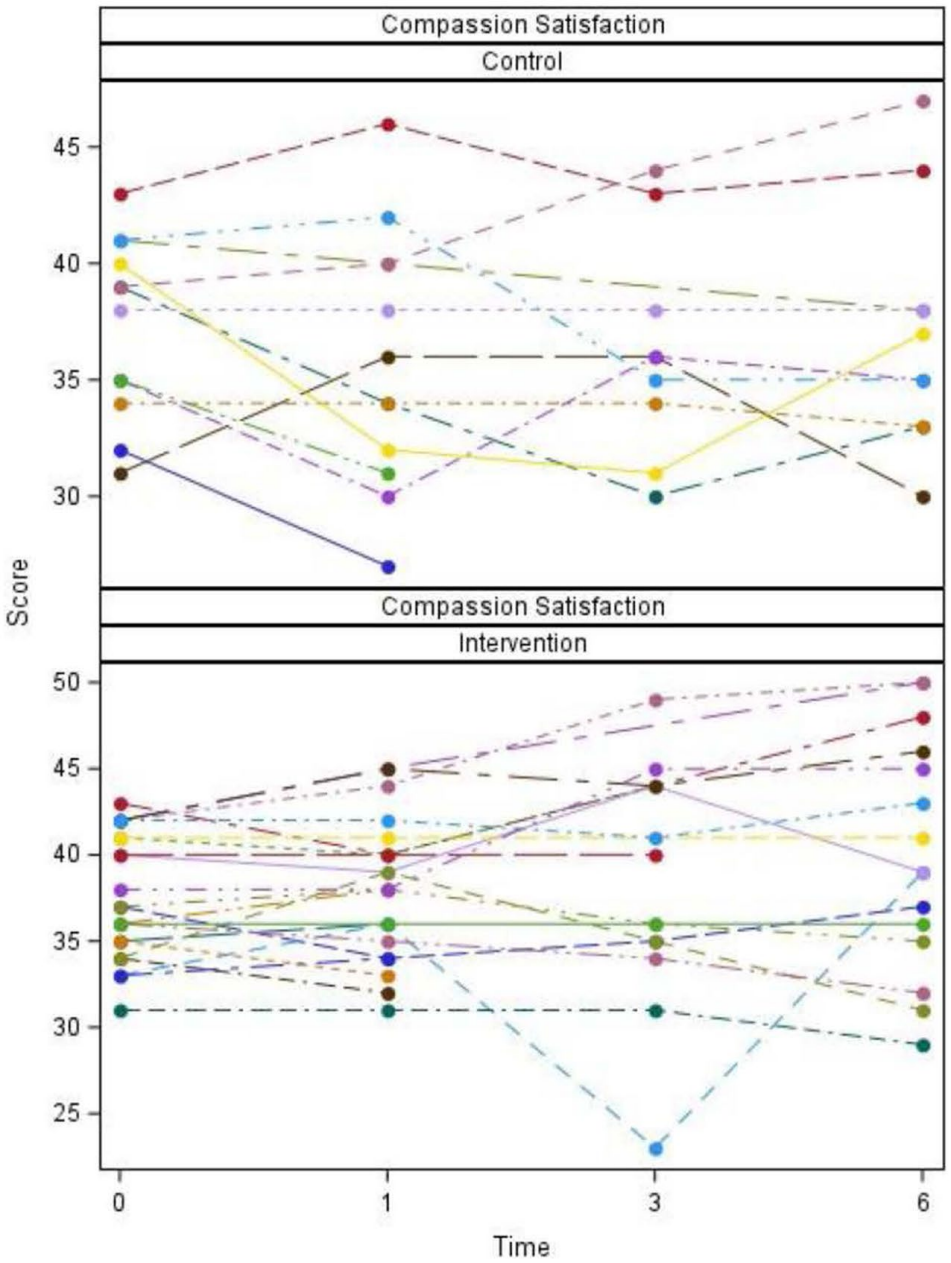
Compassion satisfaction scores by group, time, and participant.

**Figure 3:**
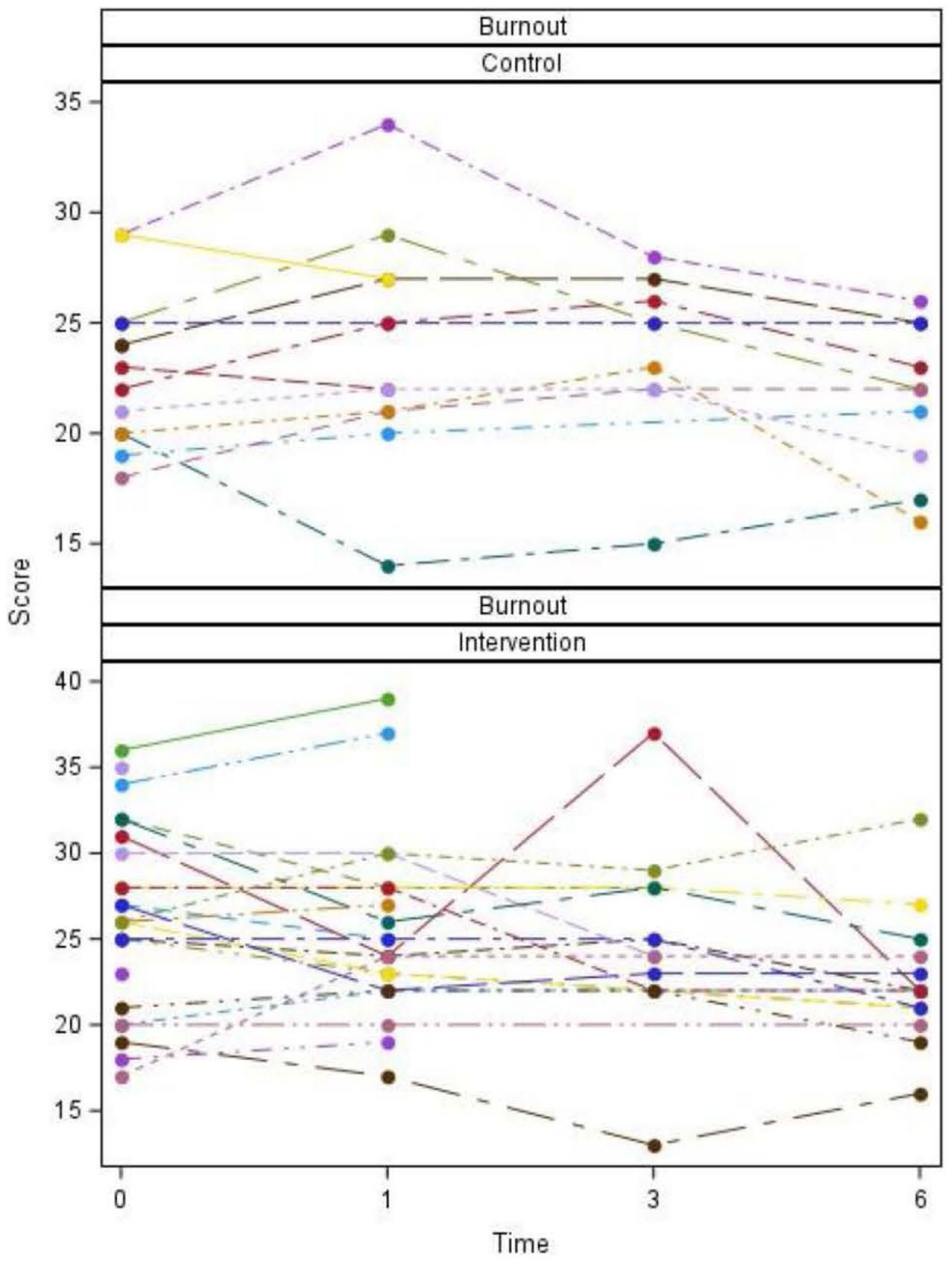
Burnout scores by group, time, and participant.

**Figure 4:**
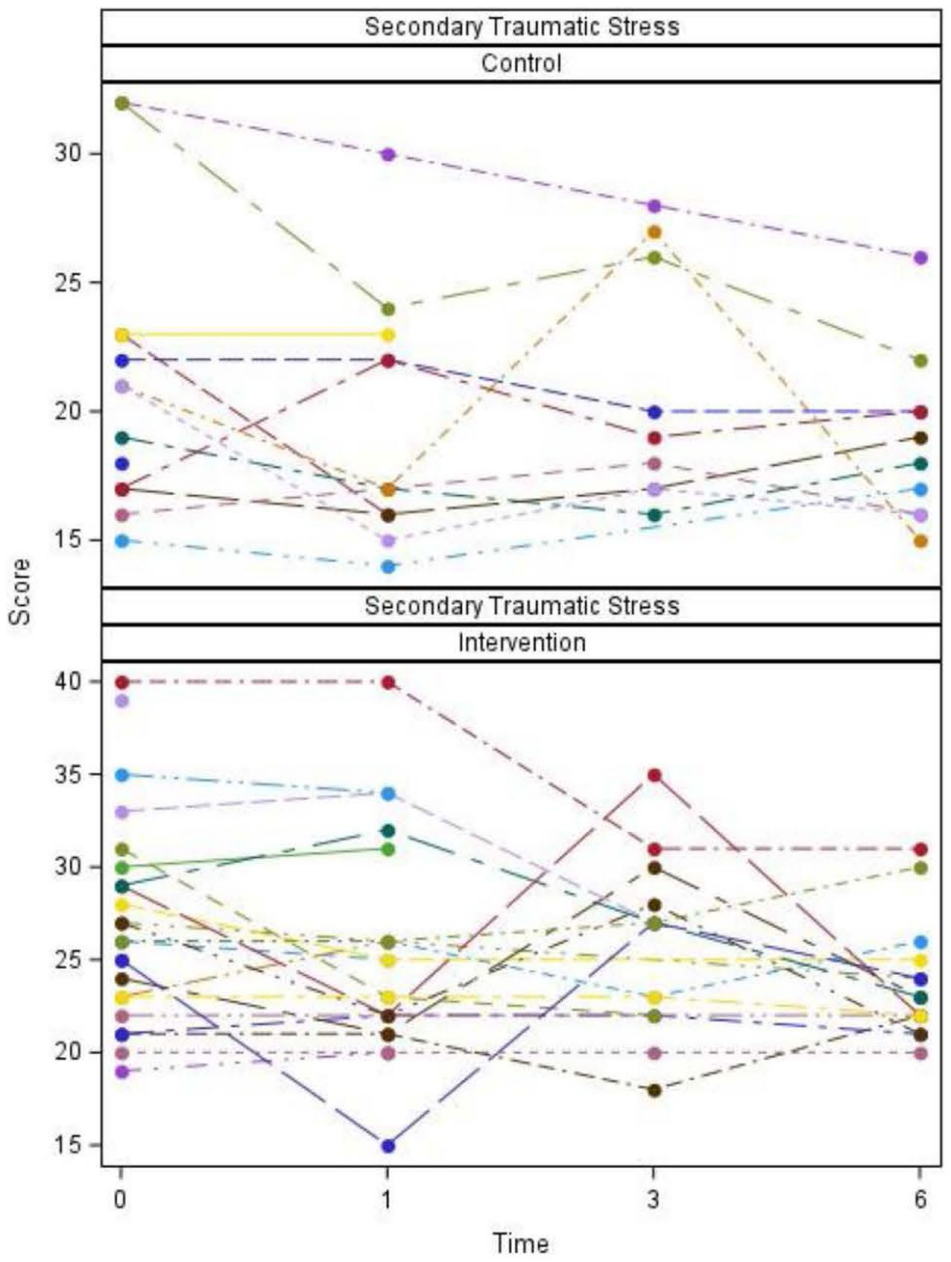
Secondary traumatic stress scores by group, time, and participant.

**Figure 5:**
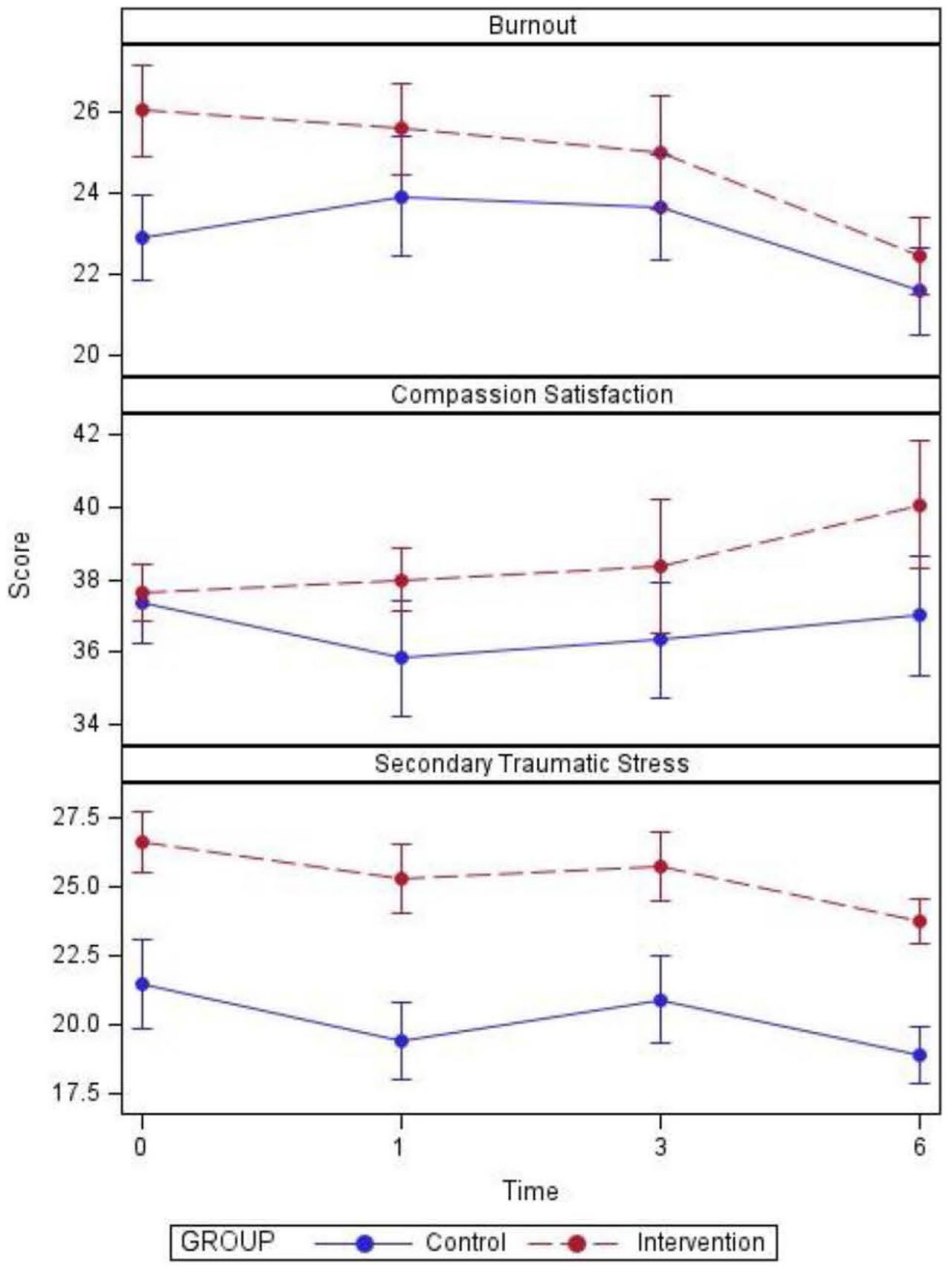
Mean ± SE PROQOL scores by group and time.

Plots of scores over time for individual participants and summarized overall participants as well as tables of descriptive statistics and *p*-values are listed (Figures 2–5; Tables 2, 3).

**Table 2:** ProQOL scores control vs. intervention group.

*Variable*	*Month*	*Group*	*N*	*Mean*	*EMM*	*Change*	*SD*	*Min*	*Max*	*C vs T^a^*
Compassion Satisfaction	1	Control	12	35.8	36.4	−1.5	5.5	27	46	0.184
		Intervention	22	38.0	38.2	0.4	4.0	31	45	.
	3	Control	9	36.3	36.1	−1.4	4.8	30	44	0.146
		Intervention	14	38.4	38.4	0.6	6.8	23	49	.
	6	Control	10	37.0	36.5	−1.1	5.2	30	47	0.051
		Intervention	15	40.1	39.5	1.8	6.8	29	50	.
Burnout	1	Control	12	23.9	25.1	1.0	5.1	14	34	0.569
		Intervention	22	25.6	24.4	−0.5	5.2	17	39	.
	3	Control	9	23.7	24.9	1.0	3.9	15	28	0.600
		Intervention	14	25.0	24.1	−0.8	5.3	13	37	.
	6	Control	10	21.6	23,1	−0.7	3.4	16	26	0.669
		Intervention	15	22.5	22.5	−2.2	3.7	16	32	.
Secondary Traumatic Stress	1	Control	12	19.4	21.3	−2.1	4.8	14	30	0.029
		Intervention	22	25.3	24.0	−1.3	5.8	15	40	.
	3	Control	9	20.9	22.5	−1.0	4.8	16	28	0.209
		Intervention	14	25.7	24.4	−1.1	4.6	18	35	.
	6	Control	10	18.9	21.0	−2.3	3.3	15	26	0.155
		Intervention	15	23.7	22.9	−2.1	3.2	20	31	.

Change, Change from Baseline. EMM, estimated marginal mean adjusted for baseline and participants with missing values. ^a^LMM with baseline covariate.

**Table 3:** Sensitivity analysis: ProQOL score change from baseline; control vs. intervention group.

*Variable*	*Month*	*Group*	*N*	*Mean*	*EMM*	*Change*	*SD*	*Min*	*Max*	*p-value^a^*
Compassion Satisfaction	1	Control	12	35.8	−1.9	−1.5	5.5	27.0	46.0	0.173
	1	Intervention	22	38.0	0.4	0.4	4.0	31.0	45.0	.
	3	Control	9	36.3	−2.3	−1.4	4.8	30.0	44.0	0.142
	3	Intervention	14	38.4	0.7	0.6	6.8	23.0	49.0	.
	6	Control	10	37.0	−3.1	−1.1	5.2	30.0	47.0	0.048
	6	Intervention	15	40.1	1.8	1.8	6.8	29.0	50.0	.
Burnout	1	Control	12	23.9	1.5	1.0	5.1	14.0	34.0	0.258
	1	Intervention	22	25.6	−0.5	−0.5	5.2	17.0	39.0	.
	3	Control	9	23.7	1.4	1.0	3.9	15.0	28.0	0.319
	3	Intervention	14	25.0	−0.6	−0.8	5.3	13.0	37.0	.
	6	Control	10	21.6	1.2	−0.7	3.4	16.0	26.0	0.386
	6	Intervention	15	22.5	−2.2	−2.2	3.7	16.0	32.0	.
Secondary Traumatic Stress	1	Control	12	19.4	−0.8	−2.1	4.8	14.0	30.0	0.581
	1	Intervention	22	25.3	−1.3	−1.3	5.8	15.0	40.0	.
	3	Control	9	20.9	−0.1	−1.0	4.8	16.0	28.0	0.968
	3	Intervention	14	25.7	−0.9	−1.1	4.6	18.0	35.0	.
	6	Control	10	18.9	−0.2	−2.3	3.3	15.0	26.0	0.917
	6	Intervention	15	23.7	−2.3	−2.1	3.2	20.0	31.0	.

Change, Change from Baseline. EMM, estimated marginal mean change from baseline adjusted for participants with missing values. ^a^LMM on change values.

## Discussion

Our study intended to identify benefits of structured daily well-being practices on compassion satisfaction and compassion fatigue as indicators of professional well-being. Study participants were veterinary helpers in a 24-h emergency and specialty practice. Our results indicate an improvement in compassion satisfaction for those helpers who regularly implemented well-being practices into their daily routines throughout the six-month study period. The impact of such practices on mitigation of burnout and secondary traumatic stress was not significant in our current study. The reason(s) for the lack of documented impact of WB on BO and STS is uncertain.

While we felt it important to include all veterinary helpers (e.g., technicians/assistants, doctors, and receptionists/front end staff) in our investigation because each worker is vulnerable to compassion fatigue (*CF*), differences certainly exist in how each group is exposed to occupational stressors as well as individual experiences within the veterinary clinic. Our study was underpowered to examine differences between such subsets of helpers and how WB may benefit them uniquely. Our study was also limited to a single hospital providing 24-h care in the Mid-Atlantic region of the United States. Duplicating this work in a multi-center study would not only be valuable to confirm our finding that WB benefits compassion satisfaction but would also have value in confirming (or refuting) our finding that WB may not mitigate *CF* as previously thought. Additionally, a larger multi-center study would allow for comparisons between the subsets of helpers. Future investigations also would be enhanced if additional information on participant demographics was considered (e.g., race, gender, age, stage of career, etc.). We saw a decline in the number of participants completing surveys with a potential decline in maintenance of WB practices over the six-month duration of our study. This decline may have contributed to less significant findings within our study analysis. More importantly, investigating the ability of helpers to maintain WB over a longer period and the question of whether WB benefits are sustained over time remain undetermined within the scope of our work. Future work should not only involve multiple centers and a greater diversity of study participants but also should be expanded to include a longer duration of investigation.

Our study sought to explore the utilization and potential positive impacts on veterinary professional wellness and compassion satisfaction when three specific evidence-based, accessible strategies were encouraged to be used daily for 6 months. The well-being activities that were selected for this study were: breath work (4–7-8 breath practice supporting parasympathetic stimulation), gratitude expression through verbalization and/or journaling, and engagement with the resources provided through the mental health platform, Lyra, that is available to all associates. Certainly, other well-being practices may have different impacts or might be more readily adopted or sustained by some than by others (12–15). These three activities were selected for this study’s purpose as they are evidence-based practices (8–11, 16–18), had low barriers to accessibility and application (time, learning, cost), and provided some variety among the three practice approaches. Additionally, there was a desire for these practices to be experienced as an easy-to-integrate part of daily routine rather than ‘additional work’ honoring the existing full schedules of these professionals.

The ProQOL as a survey tool was utilized as a well-known and validated psychometric tool for measuring secondary traumatic stress, burnout, and compassion satisfaction in a wide variety of professions, including medical caregiving. Nonetheless, there may be other tools which would be better suited to objective assessment of the impact of work experiences on veterinary professionals. An updated version of the ProQOL tool, ProQOL Health,^2^ considers the unique impact of health care helpers and includes moral distress evaluation. This tool was not available when the study began but may be a helpful instrument if the study is repeated in the future. Other writers and researchers have elected to use both alternative assessment tools or more than one tool. While utilization of additional (or alternative) tools may have value when looking to characterize the condition of individuals and/or groups within veterinary medicine (or similar caring professions), when investigating the impact of an intervention (as was the purpose of our study) limiting utilization to a single validated tool which broadly assesses both compassion satisfaction and compassion fatigue is advantageous. We acknowledge, however, that additional and alternative assessment tools exist (e.g., Maslach Burnout Inventory, Mayo Clinic Physician Burnout and Wellbeing Scale, The Optum Short Form-8 Health Survey, Kessler Psychological Distress Scale, the Connor-Davidson Resilience Scale) and expanding research in this area while utilizing more than one assessment tool may enhance the knowledge obtained and further validate (or refute) our current findings.

Compassion satisfaction comes from the positive and fulfilling aspects of doing meaningful work and positively impacting the lives of others. It has been found to correlate positively with the ability of an individual caregiver to cope, learn, and grow from difficult experiences (19). Many people that enter the veterinary profession have empathic attitudes for individuals, both people and animals, who are experiencing difficulty, as well as a strong desire to assist in alleviating such difficulties. Compassion satisfaction may not be as prevalent in veterinary helpers as would be expected given the opportunities to perform meaningful work. Holowaychuk and Lamb (20) found that veterinary emergency care providers had lower satisfaction when compared to a historical cohort of human healthcare providers. These researchers used the Maslach Burnout Inventory (MBI) Human Services Survey for Medical Personnel rather than the PROQOL and as such satisfaction was assessed in terms of workers’ feelings of Personal Accomplishment(s).

However, Ouedraogo et al. (21) did utilize ProQOL in their research which focused on a larger group of workers but was limited to veterinarians. The authors’ focus was a descriptive analysis of potential factors contributing to ProQOL scores and/or differences when considering gender, time since graduation, income, debt, and nature of the veterinary work. In contrast to the population studied by Ouedraogo, our much smaller study included veterinary helpers who were diverse in their work roles rather than being limited to only veterinarians. Within the population of veterinarians surveyed, scores for CS (mean 36.1), BO (mean 26.7) and STS (mean 22.6) were similar to those found at baseline in our study population. Interest in investigating associated factors that may contribute to ProQOL scores and describing the prevalence of low CS or high BO and STS remain a predominant focus of a growing number of veterinary studies seeking to characterize well-being within veterinary professionals across the globe. Macia et al. (5) utilized ProQOL and additional tools to assess both well-being and social supports for a large group of veterinarians in Spain. Investigators included personal demographics (i.e., gender, relationship status, domicile status, age, etc.) and solicited additional information about substance use (e.g., alcohol and/or prescription and non-prescription drugs). Mean ProQOL scores were again similar for CS (34.4) but were higher for BO (29.4) and STS (25.9). This trend was consistent with other studies that noted a lower professional satisfaction and decreased well-being in Spanish veterinarians compared with veterinarians in other countries, a difference which has been linked to lower salaries.

Dr. Stamm, one of the contributors to the development of the original and subsequent version of the ProQOL survey, has concluded that higher levels of compassion satisfaction involve “employing self-care strategies that manage stress and defend against compassion fatigue.” Additionally, to support the important work of caring with both energy and compassion, individualized and team practices that improve individual helper’s quality of life and highlight the rewarding aspects of caregiving work continually need to be developed and assessed. Thus, it makes sense that study participants who maintained WB over the six-month duration of our study, had significant improvements in their CS scores.

The concept of compassion fatigue was first introduced in 1995 by Dr. Charles Figley. It was in Figley’s 1995 publication, *Compassion Fatigue: Coping with Secondary Traumatic Stress* (22), that the term “compassion fatigue” was coined and described compassion fatigue as resulting “from stress working in traumatic situations or witnessing others’ distress.” Certainly, within veterinary medicine (and emergency and critical care work in particular), avoiding traumatic situations or others’ distress is impractical. However, there may be other factors that can be influenced within veterinary institutions and helpers’ work environments. Prior work has identified numerous factors that may contribute to BO and STS, including mental health stigma, work demand, financial stress (such as debt-to-income ratio), challenging client interactions, and increased moral distress associated when decreased hours and/or staff are perceived to compromise availability of and quality of medical care for veterinary patients. Additional variables associated with *CF* (burnout specifically) include “perceptions of an unmanageable workload, lack of control over work, little reward (recognition) for work, or an unfair allocation of resources at work (20)” (20, 21, 23, 24).

There are some prior research efforts (25) that suggest that the same stress management (WB) practices that benefit CS in individuals are also likely to decrease *CF.* In our study, participant BO and STS did not improve despite there being a significant increase in CS. Similar studies which had focused on the impact of interventions (e.g., daily well-being practices) on ProQOL scores in helpers within the human healthcare industry, documented benefits to both CS and to *CF* (11). While the results of our study may reflect true limitations of the selected WB, it is possible that the lack of significant results on decreasing BO and STS underscores the need to consider a broader approach than the practices themselves. Connection to supportive community and cultivation of a person-first culture is likely essential in mitigating or preventing elements of compassion fatigue in veterinary environments.

As previously stated, while a number of studies have identified the significant incidence of compassion fatigue within veterinary helpers (especially when compared to professionals within human healthcare) (20, 21, 23, 24), studies looking at interventions in veterinary helpers are lacking (26, 27). While this expanding body of research related to veterinary workers’ well-being is a significant contribution to further exploring the challenges compassion fatigue present to our industry, the industry and profession lack the knowledge, and certainly therefore a consensus, on strategies to mitigate the prevalence and impact of compassion fatigue on individuals, teams, and the profession at large.

Possible strategies to mitigate compassion fatigue and burnout have been discussed in the work of other veterinary researchers (25). A (more) manageable workload with control over how work is done, worker experiences of fairness and/or equity, positive rewards, and meaningful acknowledgement have been identified to positively influence veterinary helper stress and decrease some of the contributors to professional burnout. Identification and alignment of individual and of organizational values supports a culture of shared mission and purpose. When the holistic wellness of the helpers is supported by the organization and by the workplace culture, there is an increase in helper engagement, collaboration, and quality of medical care. Equally important impacts include the resulting decreases in attrition, patient medical errors, and social stigma around the mental health challenges which may occur in caregiving environments (28).

We propose that the subjective and narrative findings of our study importantly highlight the potential significance of workplace environment, culture, psychological safety and congruent values. Participants adopted informal “buddy systems” and shared that workplace interventions and resources (e.g., ability to utilize a quiet space such as the “cozy corner”) had positive impacts that extended beyond the hospital setting and impacted practices at home with extension to individuals within their family (Appendix 4). These findings align with commonly described signs of compassion satisfaction for individuals including the experience of fulfillment, achievement, inspiration, hope, and gratitude.

Our study demonstrates that veterinary helpers will embrace the incorporation of WB into their individual daily activities with perceived and proven benefit to CS and thus well-being. However, our participants also voiced challenges and impediments to completing WB (i.e., time scarcity and fatigue) which we would argue are inherent to their work environment and likely also inherent to the culture of the veterinary profession itself. In fact, the larger context within which the health and well-being of veterinary helpers becomes compromised may explain the limited benefits of WB on BO and STS. Steffey et al. have stated this most eloquently, “The issue of veterinary burnout will not be adequately addressed if the profession continues to rely predominantly on the self-care practices of individual veterinarians. More foundational change in veterinary education and practice systems will be required to ensure the sustainability of the profession under current and future societal conditions.” (25).

We hope our findings will motivate others to pursue similar work and encourage further research, looking at larger or more diverse teams and encompassing intervention(s) and assessment(s) in more than one study center. While we assume that WB (if sustained) will have long-standing benefit to helpers (especially in the bolstering of compassion satisfaction), research looking at whether WB and its benefits can be sustained over 12 months and longer and what factors may foster sustained WB is warranted.

We are inspired by the findings and opportunities identified in the current study – both within the data itself and the experiences and observations of the study participants. Additionally, the context of the study provides further evidence for the importance of communities and cultures of support for the adoption, practice, and maintenance of well-being practices. We believe that the lack of a parallel significant benefit of well-being practices in the areas of burn out and secondary traumatic stress may highlight the complexity of factors contributing to compassion fatigue in veterinary medicine. Factors include occupational stressors such as the impact of moral distress and ongoing challenges within work environments, including imbalances of resources and ever-present and increasing demands of providing care. We believe our work, and the work of others, emphasizes the vital opportunity to acknowledge an important connection between culture and team well-being for both the profession and overall industry. Indeed, as colleagues and researchers are proposing in greater numbers, individual practices of well-being may provide varying degrees of benefit to veterinary helpers’ compassion satisfaction. These benefits may be temporary and short-lived, particularly with growing recognition that deep, comprehensive, and more lasting solutions warrant focusing on root cause. Thus, sustainable solutions require organizational and professional transformation of culture and construct resulting in systemic changes that reduce the magnitude and incidence of compassion fatigue, rather than attempting to address the problem after the fact. It is our hope that all stakeholders will begin to explore and tackle the necessary transformative change. In the interim, we encourage further studies to expand knowledge of best practices to bolster veterinary teams through the adoption of well-being tools. Collaborative efforts that prioritize an integrated **“**person-first” mentality would empower the necessary changes to culture and systems to foster improved lives and sustainable careers for veterinary workers.

## Data availability statement

The original contributions presented in the study are included in the article/Supplementary material, further inquiries can be directed to the corresponding author.

## Ethics statement

Ethical review and approval was not required for the study on human participants in accordance with the local legislation and institutional requirements. The participants provided their written informed consent to participate in this study.

## Author contributions

AA: Conceptualization, Data curation, Formal analysis, Funding acquisition, Investigation, Methodology, Project administration, Supervision, Writing – original draft, Writing – review & editing. DF: Conceptualization, Data curation, Investigation, Methodology, Writing – original draft, Writing – review & editing, Funding acquisition, Project administration, Supervision. SO: Conceptualization, Data curation, Funding acquisition, Investigation, Methodology, Project administration, Resources, Writing – original draft, Writing – review & editing. VR: Data curation, Investigation, Project administration, Resources, Writing – original draft, Writing – review & editing.

## Footnotes

^1^ https://proqol.org

^2^ https://proqol.org/proqol-health-1
