# Supplementary material for: The positive impact of daily well-being practices on individual veterinary professionals’ professional quality of life self-assessment scores within an emergency and specialty hospital
Source: Front Vet Sci. 2024 Oct 8;11:1381090. doi: 10.3389/fvets.2024.1381090 (PMC11496890; doi:10.3389/fvets.2024.1381090)

Appendix 1: TERMS AND DEFINITIONS UTILIZED AND CONSIDERED IN THE MANUSCRIPT AND RELEVANT TO THE SUBJECT MATTER OF "WELL-BEING"

Burnout: a syndrome conceptualized as resulting from chronic workplace stress that has not been successfully managed. It is characterized by three dimensions:

- feelings of energy depletion or exhaustion;
- increased mental distance from one's job, or feelings of negativism or cynicism related to one's job; and
- reduced professional efficacy.

Caregivers/Helpers: A person who provides direct care physically and emotionally; assists with activities of daily living. For animal caretakers, this includes:

- Access to healthy food + fresh water
- Grooming
- Bathing
- Exercise
- or otherwise care to promote and maintain the well-being of pets and other animals that are not raised for consumption

In a hospital setting, this also involves cage or enclosure management, medication management, monitoring, and internal/external advocacy.

Compassion Fatigue: broadly defined concept that can include emotional, physical, and spiritual distress in those providing care to another. Compassion fatigue is associated with caregiving where individuals are experiencing significant emotional or physical pain and suffering. See also: www.compassionfatigue.org; "Compassion fatigue is the over identification with another individual's emotional pain. It starts with stress from the intense desire to meet the needs of others and results in the complete inability to relate to colleagues or clients."

Compassion Satisfaction: positive and fulfilling consequence of helping behavior/meaningful work. Compassion satisfaction is the pleasure you derive from helping, positive feelings we have for colleagues and a good feeling resulting from the ability to assist others and contribute.

Emergency & Critical Care: provides immediate evaluation, stabilization, and treatment of pets with emergent or serious conditions; the team may consist of emergency and specialty veterinary workers

Helper(s): an individual who provides care for or support for others in either a professional work capacity or as a volunteer. Commonly referring to health care workers (including mental health care workers) but applicable to the veterinary profession as well as many other caregivers (i.e. teachers, parents, first responders, etc.). For our manuscript, the term "helpers" will be used as a synonym for any of the veterinary workers studied. See also ***Caregivers.***

Holistic Wellness: an approach to life that considers multidimensional aspects of wellness; it comprises eight mutually co-dependence dimensions: emotional, physical, occupational, social, spiritual, intellectual, environmental, and financial.

Mental health: a state of mental well-being that enables people to cope with the stresses of life, realize their abilities, learn well and work well, and contribute to their community; underpinning our individual and collective abilities to make decisions, build relationships and shape the world we live in and It exists on a complex continuum, which is experienced differently from one person to the next, with varying degrees of difficulty and distress and potentially very different social and clinical outcomes.

Moral Distress: Moral distress is said to occur when one has made a judgment but is unable to act upon it. In veterinary medicine this includes complicity in perceived wrongdoing, lack of power, ethical strain, as well as repeated heightened experiences within levels of patient, team and system.

Occupational stressors: Related to work-related stress and the response people may have when presented with work demands and pressures that are not matched to their knowledge and abilities and which challenge their ability to cope. This includes lack of control over processes, poor work design, poor management, unsatisfactory working conditions, and lack of support from colleagues and supervisors. See also ***Psychological Stressors.***

Practice Related Stressors: occupational stressors that are related to veterinary practice environments and/or the provision of veterinary care including but not limited to demands of practice; practice management responsibilities; making professional mistakes; client complaints; dealing with personal, staff, or client grief; client expectations of being expert in all veterinary subject areas; animal deaths; competition with other veterinary practices; ethical challenges; fear of malpractice litigation; educational debt; poor social support; unclear management and work role; and lack of participation in decision-making.

Professional Quality of Life: Understanding the positive and negative aspects of helping those who experience trauma and suffering can improve your ability to help them and your ability to keep your own balance.

Professional Well-Being: relates to all aspects of working life, from the quality and safety of the physical environment, to how workers feel about their work, their working environment, the climate at work and work organization. It also includes job satisfaction, finding meaning in work, feeling engaged and fulfilled with work, having a high-quality working life, and professional fulfillment.

Psychological Safety: can be described by the absence of interpersonal fear; psychological safety is a shared belief held by members of a team that it's OK to take risks, to express their ideas and concerns, to speak up with questions, and to admit mistakes — all without fear of negative consequences. It can also be defined by these four stages:

1. **Inclusion Safety** – members feel safe to belong to the team. They are comfortable being present, do not feel excluded, and feel like they are wanted and appreciated.
2. **Learner Safety** – members can learn through asking questions. Team members here may be able to experiment, make (and admit) small mistakes, and ask for help.

3. **Contributor Safety** – members feel safe to contribute their own ideas, without fear of embarrassment or ridicule. This is a more challenging state, because volunteering your own ideas can increase the psychosocial vulnerability of team members.
4. **Challenger Safety** – members can question others' (including those in authority) ideas or suggest significant changes to ideas, plans, or ways of working.

Psychological Stress: a particular relationship between the person and the environment that is appraised by the person as taxing or exceeding his or her resources and endangering his or her well-being.

Psychological stressors in the veterinary profession include, but are not limited to:

- work/life balance
- schedule management
- financial strain
- Aesculapian authority
- client demands/expectations
- high emotionality in interpersonal relationships, workplace disharmonies and intersectional microaggressions
- exposure to abuse, violence, injuries, chronic and terminal illness management
- and ethical dilemmas regarding treatment options including euthanasia

Secondary Traumatic Stress: refers to the distress and emotional disruption resulting from continued and cumulative contact, or after single exposure, with individuals who have directly experienced trauma. Work that involves witnessing a great deal of suffering, by observing or listening to narratives of trauma, can result in lasting emotional distress bringing about feelings of frustration, a sense of loss of control, and decreased morale.

Veterinary professionals: We recognize that the term "veterinary professionals" may mean different things to different people and may be unclear and/or limiting (if not exclusionary). We consider many individuals to be veterinary professionals and would define the term as an individual working within the veterinary profession in any of a variety of roles or activities including reception and client care workers (commonly referred to as customer service coordinators), veterinary assistants, veterinary technicians (certified or pursuing licensure or certification), team supervisors, hospital managers and/or administrators and veterinarians. However, due to the potential for unclear, confusing, or narrow conceptualization of who "is" a veterinary professional, we elect to refer to "**veterinary workers**" throughout much of our manuscript but do consider the term synonymous with the term "**veterinary professionals**".

Veterinary Medical Professionals can include, but are not limited to:

- *Animal care*:* Veterinarians, Veterinary Technician Managers, Veterinary Technicians, Veterinary Assistants, Kennel Assistant
- *Front of house support*: Client service manager, Client service coordinators
- *Back of house support:* on-site administrators including hospital administrator, technician supervisors, practice manager, assistant practice manager

Practice dependent regarding level of involvement in patient care and leadership

Veterinary team: When considering veterinary workers or veterinary professionals as a group; the term "***veterinary team***" should be considered synonymous with these groups or terms.

Veterinary Workers: To avoid the potential for unclear, confusing or narrow conceptualization of who "is" a veterinary professional and who contributes to the individual and collective work of veterinary teams, we elect to refer to "**veterinary workers**" throughout much of our manuscript and consider the term synonymous with the term "**veterinary professionals**".

Well-being: encompasses quality of life, as well as the ability of people and societies to contribute to the world in accordance with a sense of meaning and purpose; observed by the extent to which they are resilient, build capacity for action, and are prepared to transcend challenges

Well-being practices: practices that incorporate concepts of self-care (and self-compassion) to create, support, or enhance well-being. Practices can be focused on individuals, groups or teams, or cultures or environments. Examples include practices of gratitude, meditation, breathing exercises, other mindfulness practices or exercises, and mental health supports (e.g. individualized therapy, group therapy, or support groups).

Appendix 2: Professional Quality of Life Scale (ProQOL) Self-Assessment Questionnaire

COMPASSION SATISFACTION AND COMPASSION FATIGUE (PROQOL) VERSION 5 (2009)

When you *[help]* people you have direct contact with their lives. As you may have found, your compassion for those you *[help]* can affect you in positive and negative ways. Below are some questions about your experiences, both positive and negative, as a *[helper]*. Consider each of the following questions about you and your current work situation. Select the number that honestly reflects how frequently you experienced these things in the *last 30 days*.

**1=Never**	**2=Rarely**	**3=Sometimes**	**4=Often**	**5=Very Often**

1. I am happy.
2. I am preoccupied with more than one person I *[help]*.
3. I get satisfaction from being able to *[help]* people.
4. I feel connected to others.
5. I jump or am startled by unexpected sounds.
6. I feel invigorated after working with those I *[help]*.
7. I find it difficult to separate my personal life from my life as a *[helper]*.
8. I am not as productive at work because I am losing sleep over traumatic experiences of a person I *[help]*.
9. I think that I might have been affected by the traumatic stress of those I *[help]*.
10. I feel trapped by my job as a *[helper]*.
11. Because of my *[helping]*, I have felt "on edge" about various things.
12. I like my work as a *[helper]*.
13. I feel depressed because of the traumatic experiences of the people I *[help]*.
14. I feel as though I am experiencing the trauma of someone I have *[helped]*.
15. I have beliefs that sustain me.
16. I am pleased with how I am able to keep up with *[helping]* techniques and protocols.
17. I am the person I always wanted to be.
18. My work makes me feel satisfied.
19. I feel worn out because of my work as a *[helper]*.
20. I have happy thoughts and feelings about those I *[help]* and how I could help them.
21. I feel overwhelmed because my case [work] load seems endless.
22. I believe I can make a difference through my work.
23. I avoid certain activities or situations because they remind me of frightening experiences of the people I *[help]*.
24. I am proud of what I can do to *[help]*.
25. As a result of my *[helping]*, I have intrusive, frightening thoughts.
26. I feel "bogged down" by the system.
27. I have thoughts that I am a "success" as a *[helper]*.
28. I can't recall important parts of my work with trauma victims.
29. I am a very caring person.
30. I am happy that I chose to do this work.

Appendix 3: MENU OF WELL-BEING PRACTICES FOR STUDY PARTICIPANTS: Study participants had the option to choose from one (or a combination of) the below three well-being practices. The impact of these interventions on participants' well-being was assessed via serial assessment of participants' ProQOL scores.

1. **Utilization of LYRA mental health platform:** Lyra is a web/app-based mental health platform which provides instant access to diverse resources. Available resources include individualized support and care ranging from self-guided programs and mental health coaching to therapy and medication management. The care options are intended to accommodate personal preferences and lifestyles with compassionate and culturally responsive care. The tool includes 24/7 access to an in-house concierge care navigator team to help guide users in their mental health journey.

2. **Daily practice of gratitude:** a positive emotional reaction or personal meditation in response to the receipt of a gift or benefit from someone or something; used to grow a skill set in grounding and reframing. Gratitude practices require private, intentional, reflection time and can focus on sensory stimulation or any number of grounding techniques focusing on the present moment. The utilization of these practices considers the attention paid to minute details, the emotional response and how we use these experiences to influence future oriented cognition.

3. **Practice of paced breathing exercises:** Breathing exercises generally involve slow, deep, diaphragmatic breathing that reduces central sympathetic activity and facilitates the relaxation response. Primary exercise and instruction emphasized 4-7-8 breathing but resources and education for other breathing exercises were provided.

Appendix 4: Sampling of subjective data from "Wellness Wednesdays", reflective of participant experiences, well-being practices, and commentary.

"I did written gratitude every day and even included my [child]!"
"While at work, I step away to walk outside to feel the sun on my face, take my mask off, and breath in the smells of spring emerging."
"It has now become habit to do without even thinking about it when I am stressed. Also use it at bedtime and before work."
"I now have a schedule for yoga twice a week and my instructor and I have added moments of gratitude at the end of every session."

Utilization of a breathing app on smart watches
Incorporating breathing exercises into a child's bedtime and FALLING ASLEEP
Intentional use of breathing exercises to help transition to sleep
Use of breathing exercises to help with anxiety at work and improve focus
"at work I added 10 minutes to the end of every lunch break to relax in the cozy corner before returning to work."
Taking 10 minutes to eat on a lunch break and 20 minutes for time in "cozy corner"

"I've combo's 4-7-8, gratitude, and micro breaks at work. Work has been easier this past week"
Trying a different breathing technique when 4-7-8 breathing seemed to become less effective
Improvements in self-esteem ('*seeing more value in myself*'), improvements in or gratitude for resiliency. Deliberate *"taking time for [self] to decompress and relax"*.
A participant shared use of deep breathing then 4-7-8 breathing as well as 'body scanning' at work and use of the Lyra resources-- *"Thankful for the many outlets I have to reduce my anxiety, especially at work."*

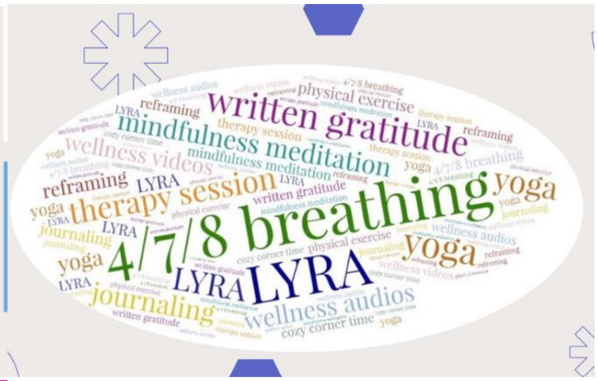

Appendix 5: Sampling of flyers utilized for recruitment, education, reminders of well-being practices and reminders for completion of ProQOL surveys and "check-ins" with well-being educator

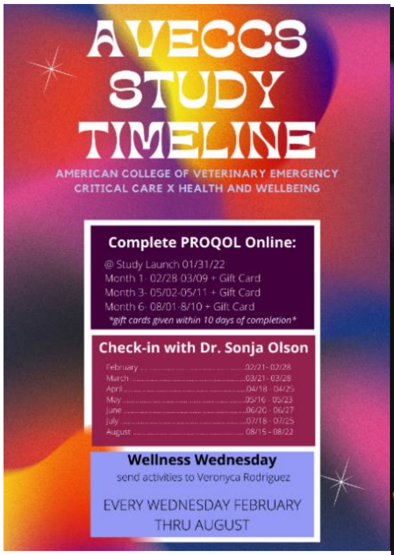

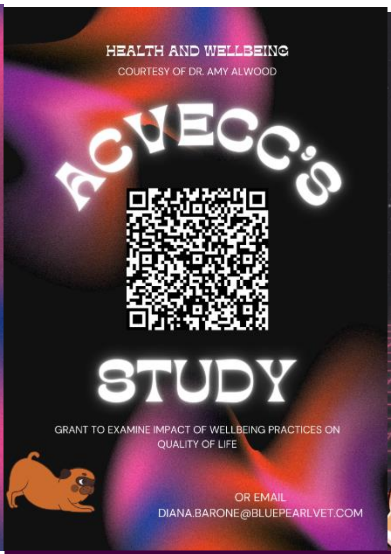

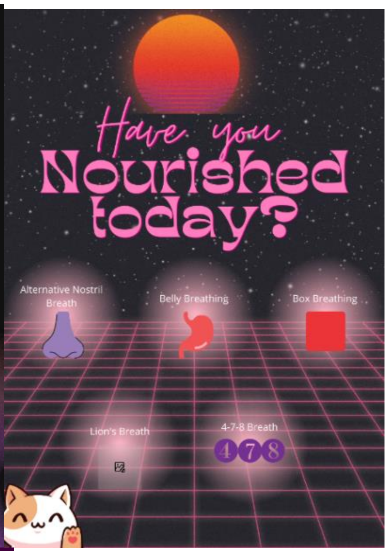

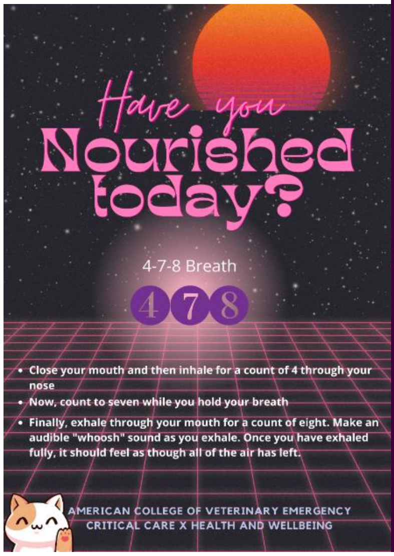

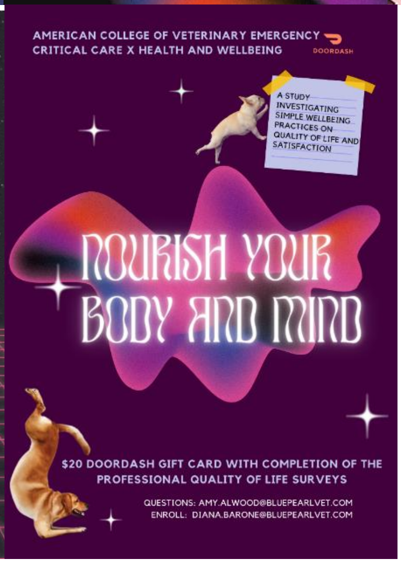

Supplement: Supplementary file 1 [file Presentation_1.pdf]
